# Virotyping and genetic antimicrobial susceptibility testing of porcine ETEC/STEC strains and associated plasmid types

**DOI:** 10.3389/fmicb.2023.1139312

**Published:** 2023-04-17

**Authors:** Nick Vereecke, Sander Van Hoorde, Daniel Sperling, Sebastiaan Theuns, Bert Devriendt, Eric Cox

**Affiliations:** ^1^Laboratory of Virology, Department of Translational Physiology, Infectiology and Public Health, Faculty of Veterinary Medicine, Ghent University, Merelbeke, Belgium; ^2^PathoSense BV, Lier, Belgium; ^3^Laboratory of Immunology, Department of Translational Physiology, Infectiology and Public Health, Faculty of Veterinary Medicine, Ghent University, Merelbeke, Belgium; ^4^CEVA Santé Animale, Libourne, France

**Keywords:** pathogenic *Escherichia coli*, edema disease, nanopore sequencing, antibiotic resistance, genomics

## Abstract

**Introduction:**

Enterotoxigenic *Escherichia coli* (ETEC) infections are the most common cause of secretory diarrhea in suckling and post-weaning piglets. For the latter, Shiga toxin-producing *Escherichia coli* (STEC) also cause edema disease. This pathogen leads to significant economic losses. ETEC/STEC strains can be distinguished from general *E. coli* by the presence of different host colonization factors (e.g., F4 and F18 fimbriae) and various toxins (e.g., LT, Stx2e, STa, STb, EAST-1). Increased resistance against a wide variety of antimicrobial drugs, such as paromomycin, trimethoprim, and tetracyclines, has been observed. Nowadays, diagnosing an ETEC/STEC infection requires culture-dependent antimicrobial susceptibility testing (AST) and multiplex PCRs, which are costly and time-consuming.

**Methods:**

Here, nanopore sequencing was used on 94 field isolates to assess the predictive power, using the meta R package to determine sensitivity and specificity and associated credibility intervals of genotypes associated with virulence and AMR.

**Results:**

Genetic markers associated with resistance for amoxicillin (plasmid-encoded TEM genes), cephalosporins (*ampC* promoter mutations), colistin (*mcr* genes), aminoglycosides (*aac(3)* and *aph(3)* genes), florfenicol (*floR*), tetracyclines (*tet* genes), and trimethoprim-sulfa (*dfrA* genes) could explain most acquired resistance phenotypes. Most of the genes were plasmid-encoded, of which some collocated on a multi-resistance plasmid (12 genes against 4 antimicrobial classes). For fluoroquinolones, AMR was addressed by point mutations within the ParC and GyrA proteins and the *qnrS1* gene. In addition, long-read data allowed to study the genetic landscape of virulence- and AMR-carrying plasmids, highlighting a complex interplay of multi-replicon plasmids with varying host ranges.

**Conclusion:**

Our results showed promising sensitivity and specificity for the detection of all common virulence factors and most resistance genotypes. The use of the identified genetic hallmarks will contribute to the simultaneous identification, pathotyping, and genetic AST within a single diagnostic test. This will revolutionize future quicker and more cost-efficient (meta)genomics-driven diagnostics in veterinary medicine and contribute to epidemiological studies, monitoring, tailored vaccination, and management.

## 1. Introduction

Enteric colibacillosis is the most common cause of secretory diarrhea in pigs, causing major problems in suckling and post-weaning piglets. Overall, infections with pathogenic *E. coli* result in significant economic losses due to its high morbidity and increased mortality (USDA, 2016; Luppi, 2017). Enterotoxigenic *Escherichia coli* (ETEC) infect the small intestinal tract, which results in neonatal diarrhea (ND) and post-weaning diarrhea (PWD) in suckling and post-weaning piglets, respectively. If an *E. coli* strain expresses Shiga-toxin (STEC), nursery pigs show neurological symptoms of edema disease (ED) (Jahanbakhsh et al., 2016; Fairbrother and Nadeau, 2019). In general, ETEC/STEC strains can be distinguished from commensal *E. coli* strains by the presence of virulence genes, which are classified in adhesins and enterotoxins. The most common fimbrial genes encode for F4, F5, F6, F18, or F41 proteins. While F4 fimbriae, encoded by the *fae(CDEGHIJ)* operon, are commonly isolated from PWD and ND, F18 fimbriae (*fed(ABCEF)* operon) are mostly associated with PWD and ED. The F5, F6, and F41 fimbrial proteins are generally linked to ND (Dubreuil et al., 2016; Luppi, 2017; Fairbrother and Nadeau, 2019). Also, a non-fimbrial protein (AIDA) is known to play a role in adhesion (Mainil et al., 2002). Adhesins allow the initial establishment of an infection, followed by the expression of either one or a combination of enterotoxins. These include the heat-labile (e.g., LTI and LTII) and heat-stable enterotoxins (e.g., STa, STb, and EAST1). Based on the combination of adhesins and enterotoxins, different *E. coli* virotypes have been described (Fairbrother and Nadeau, 2019). Still, some strains lack the presence of a known (non-)fimbrial adhesin (Frydendahl, 2002; Jahanbakhsh et al., 2016). Most of these virulence genes are encoded on mobile genetic elements (MGE), such as plasmids, bacteriophages, or pathogenicity islands. A wide variety of virulence and AMR-mediating plasmids have been described, which are usually classified into incompatibility groups (Novick, 1987; Mainil et al., 1992; Rozwandowicz et al., 2018). The most often used typing plasmid scheme relies on the replicon (rep) type. This is often combined with the relaxase type and mating pair formation (mpf) system to determine plasmid transferability (or mobilization) and host range (Robertson and Nash, 2018; Rozwandowicz et al., 2018).Also multi-replicon plasmids exist (i.e., cointegration of different plasmids), which represents an additional layer of complexity (Villa et al., 2010; Rozwandowicz et al., 2018; Wang et al., 2021a). Next to ETEC/STEC virotyping, *E. coli* strains can also be serotyped based on their surface O (polysaccharide), H (flagellar), and K (capsular) antigens. Though, this is not widely applied in diagnostics since many non-typeable pathogenic *E. coli* have been identified. Also, not all strains of a given serotype are pathogenic and not all diagnostic laboratories perform these tests routinely (Fairbrother and Nadeau, 2019). Nevertheless, serotyping is quick, cost-efficient, and allows the instantaneous identification of known pathogenic serotypes, such as the zoonotic O157:H7 (Fairbrother and Nadeau, 2006). To date, most routine diagnostics laboratories rely on the use of *E. coli* pathotyping *via* ELISA or (multiplex) PCR methods (Francis, 2002; Frydendahl, 2002). The latter is preferred as antigen-based methods require the appropriate growth conditions to assure *in vitro* expression of the tested adhesins and toxins (Fairbrother and Nadeau, 2019).

Over the last years increased acquired resistance has been reported for pathogenic *E. coli*, including resistance toward amoxicillin, paromomycin, trimethoprim, and tetracyclines (Begum et al., 2016; Luppi, 2017; Peng et al., 2022). While treatment of PWD relies on antimicrobials and electrolytes, prevention is recommended *via* vaccination of gilts/sows, piglets, or weaned pigs against ND, ED, or PWD, respectively. Also, hygienic measures are important since these *Escherichia coli* bacteria can persist in manure up to 6 months (Jones, 1980; AMCRA, 2022). Still, antimicrobial therapies for enteric colibacillosis are an important source of antimicrobial use in swine production. For ED, the prognosis is worse since the Shiga toxin has already bound receptors when neurological symptoms are presented (Fairbrother and Nadeau, 2019). Hence, antimicrobial treatment is discouraged here. Depending on local regulations, therapeutics should be chosen appropriately (i.e., pharmacokinetics) as the drugs should reach and concentrate in the intestinal tract and mucosa. Effective drugs include amoxicillin, apramycin, ceftiofur, gentamicin, trimethoprim/sulfa, oxytetracycline, and fluoroquinolones among others. Still, fluoroquinolones and cephalosporins (3^rd^ and 4^th^ generation) are considered critically important for humans, hence the swine industry is pressurized to minimize its use (AMCRA, 2020; European Medicines Agency, 2020; Kim et al., 2022). As such it is important to understand mechanisms and dissemination of these AMR genes (ARGs) (AMCRA, 2020; Hartadi et al., 2020). To date, antimicrobial susceptibility testing (AST) of pathogenic *E. coli* is most often done using culture-based methods or automated systems (Prieto et al., 2022). Though this usually requires selective cultures using blood or MacConkey agar plates to select for Enterobacteriaceae and is commonly supplemented with in-depth biochemical characterization, serotyping, and/or virotyping (Luppi, 2017). The culture-dependent and multilayered nature of ETEC/STEC diagnostics highlight its time and costly nature.

Over the last years, sequencing has become more affordable due to the availability of second- and third-generation sequencing methodologies (Goodwin et al., 2016; Wang et al., 2021b). While Illumina short-read sequencing has been the predominant player in sequencing, its use in bacterial diagnostics has not been widely implemented for diagnostics. The use of long-read nanopore sequencing has shed new light on diagnostics. As exemplified for other bacterial species, including *Mycobacterium tuberculosis* and *Mycoplasmopsis bovis*, cost-efficient and real-time (meta-)genomic sequencing represents a potential new revolution of diagnostics (Pightling et al., 2018; Theuns et al., 2018; Charalampous et al., 2019; Cabibbe et al., 2020; Bokma et al., 2021b). Nevertheless, to date, most data are limited to academic research without its wider implementation in routine diagnostic laboratories. It is however believed that the use of sequencing-based diagnostics can significantly contribute to *all-in-one* diagnostics of bacterial infections to study epidemiology, monitor spread of ARGs, tailor treatments, and address changes in management (Van Hoorde and Butler, 2018; Koutsoumanis et al., 2020). Hence, this work focused on the *all-in-one* diagnostic potential of long-read nanopore sequencing to tailor treatments of enteric colibacillosis. The generation of complete circular ETEC/STEC genomes and plasmids allowed to address predictive power of genetic markers of virulence and AMR as compared to multiplex PCR and phenotypic agar disc diffusion, respectively. In addition, our data allowed to perform in-depth characterization of MGEs involved in virulence and acquired resistance.

## 2. Materials and methods

### 2.1. Collection of Enterotoxigenic and Shiga toxin-producing *Escherichia coli* strains

Ninety-four field samples were collected between 2011 and 2022 in Belgium and The Netherlands. Fecal samples, from pigs with diarrhea, were inoculated on selective-indicative MacConkey agar plates to select for Enterobacteriaceae. A single Lactose-positive colonies was collected and streaked onto a new MacConkey agar plate, followed by identification using matrix-assisted laser desorption/ionization-time of flight mass spectrometry MALDI-TOF MS with a cut-off score > 2 for most strains. From each sample, aliquots were stored at −80°C in a 35% glycerol solution. An overview of all samples can be found in Supplementary Table S1.

### 2.2. Virotyping and phenotypic antimicrobial susceptibility testing of ETEC/STEC strains

Virotyping was performed at the laboratory of immunology (Department of Translational Physiology, Infectiology, and Public Health, Ghent University, Faculty of Veterinary Medicine), where an *in-house* multiplex PCR was run to test for F4, F18, LT, STa, STb, and Stx2e (Casey and Bosworth, 2009). In short, isolated ETEC/STEC strains were grown on a blood agar plate, after which a colony multiplex PCR was performed. Positivity was verified with gel electrophoresis by analyzing resulting PCR amplicon lengths corresponding to specific genes. In each PCR, two positive controls were included; the GIS26 (F4:LT:STa:STb) and F107/86 (F18:Stx2e) reference strains. Growth on blood agar plates also allowed to address hemolytic features of each strain. Phenotypic evaluation of antimicrobial susceptibility of all strains was performed at DGZ Vlaanderen. The 18 antimicrobial compounds (cefalexin, ceftiofur, amoxicillin, amoxicillin-clavulanic acid (CA), cefquinome, tetracycline, doxycycline, flumequine, enrofloxacin, marbofloxacin, gentamicin, apramycin, kanamycin, trimethoprim-sulfamethoxazole, florfenicol, spectinomycin, colistin, and paromomycin) were selected based on their antibiotic class relevance to the field and importance for human health. The specific norm AFNOR NF U47-107 was used to determine the antibiotic susceptibility and breakpoint according to the guidance of Societé Française de microbiologie (CA-SF). Mueller-Hinton (MH) agar plates (Bio-Rad) were inoculated with the strain adjusted to the density of 0.5 McFarland turbidity, the different paper discs (Axonlab) were placed on the surface of the inoculated agar plate. After aerobic incubation at 37°C for 18 h ± 2 h the inhibition diameter was measured. Resulting disc diameters (mm) were used to determine the epidemiological cut-off (ECOFF) value for each antimicrobial drug using the normalized resistance interpretation (NRI) tool (Kronvall, 2010). This allowed to determine a wild-type (WT) and non-wild type (non-WT) population for each antimicrobial drug. For colistin the antimicrobial susceptibility was performed by E-test (bioMérieux). For colistin, strains were classified as sensitive when the E-test showed growth at ≤2 μg.mL^−1^ as described/reviewed before (Vasoo, 2017; Pogue et al., 2020). The *E. coli* ATCC 25922, *Staphylococcus aureus* ATCC 25923, and *Pasteurella multocida* ATCC 43137 reference strains were used as quality control. A detailed overview of multiplex PCR and AST results is given in Supplementary Table S2 and Supplementary Figure S1.

### 2.3. Long-read whole-genome nanopore sequencing

For long-read nanopore sequencing, an aliquot of each culture was thawed and inoculated in 10 mL LB medium for overnight incubation. The next day, 4 mL was centrifuged at 16,000 × *g* to collect all bacterial cells. Pellets were resuspended in 250 μL dPBS prior to the isolation of high molecular weight (HMW) DNA using the ZymoBIOMICS DNA MiniPrep kit (Zymo Research) at the PathoSense laboratory. Manufacturer’s instructions were followed, with the addition of a 30-min Proteinase K treatment (20 μg.μL^−1^; Promega) after cellular disruption using a TissueLyser (Qiagen) for twice 5 min at 30 oscillations per minute. Quality of the HMW DNA was verified using a NanoDrop Spectrophotometer. If A_260_/A_230_ or A_260_/A_280_ were below 1.7, DNA was subjected to an additional clean-up using CleanNGS (CleanNA) magnetic beads in a 1:1 ratio. High quality HMW DNA was subjected to sequencing using 400 ng DNA input per isolate in a rapid library preparation (SQK-RBK004; ONT). A maximum of 10 isolates were multiplexed on a single R.9.4.1 flow cell. Sequencing was done on a GridION device (ONT), allowing raw data demultiplexing and collection in the MinKNOW software (ONT). To evaluate nanopore sequencing quality and accuracy, DNA from an *E. coli* ATCC 25922 strain was isolated and sequenced in the same workflow as described before. This was done in triplicate to address final genome completeness and accuracy as determined against the *E. coli* K ATCC 25922 genome (NZ_CP009072.1) using CheckM (based on 1,212 genes from 27 *E. coli* reference genomes) and pomoxis (ONT), respectively (Vereecke et al., 2020, 2023; Bokma et al., 2021a). An overview of sequencing output and coverage can be found in Supplementary Table S1.

### 2.4. Generation of complete circular and accurate ETEC/STEC genome assemblies

Resulting demultiplexed fast5 files were subjected to Bonito base calling v0.4.0 (ONT R&D base caller), using its *default* model (dna_r9.4.1@v3.3 model) on the Ghent University HPC Tier 2 GPU cluster Joltik using 1x GPU. The fastq files per isolate were used in an *in-house* bacterial whole-genome assembly pipeline using the Trycycler pipeline v0.5.3 (Wick et al., 2021a). In short, reads were filtered using filtlong v0.2.1 (--min_length 1,000 --keep_percent 95)^1^ prior to subsampling into 10 subsamples [--min_read_depth 50 --count 10 --genome_size 5 M (Wick et al., 2021a)]. Each subsampled fastq file was used to perform an independent initial genome assembly using either flye v2.9 (Kolmogorov et al., 2019), raven v1.8.1 (Vaser and Šikić, 2021), miniasm_and_minipolish.sh v0.3,^2^ or wtdbg2 v1.12 (Ruan and Li, 2020) as instructed on the Trycycler manual page. Final assemblies were used as input for Trycycler to generate consensus genomes using *default* settings. This includes the removal of contigs with coverage below 10% of the median sample sequencing coverage, clustering, reconciling, multiple sequence alignment, read partitioning, and the generation of complete circular genome and plasmid consensuses. The latter were subjected to final read mapping and polishing using minimap2 v2.20 (Li, 2018) and medaka v1.5.0 (ONT), respectively. If no consensus assembly could be obtained from Trycycler (e.g., too contiguous assemblies), flye assemblies were used for mapping and polishing. These strains were indicated with an F at the end of the strain name. Contigs were classified to chromosomes and plasmids using ViralVerify v1.1.^3^ Completeness and accuracy of final consensus genomes was assessed using ribosomal multi-locus sequence typing (rMLST) (Jolley et al., 2018) and CheckM v1.1.0 (Parks et al., 2015). A genome was considered complete based on 1,212 marker genes from 27 *Escherichia* genomes. When all marker genes were identified, a completeness of 100% was reported. Genome QC reports and associated NCBI accession numbers can be found in Supplementary Table S1.

### 2.5. Identification of genetic virulence- and AMR-associated hallmarks

Phylogenetic inference was done using the csi phylogeny tool to obtain a single-nucleotide polymorphism (SNP) alignment of all genomes (Kaas et al., 2014). The ETEC strain 1729 was used as reference. The SNP alignment was then used to build a maximum likelihood (ML) phylogenetic tree in IQ-tree2 v1.6.1 with 1,000 ultrafast bootstraps (−bb) (Minh et al., 2020). Tree visualizations were done in iTOL (Letunic and Bork, 2021). Genomes were screened for virulence and ARG markers using Abricate v1.0.1^4^ and most recent databases of virulence [ecoli_vf: virulence factor database (vfdb)]^5^ and resistance genes [Resfinder: (Bortolaia et al., 2020)]. Outputs are summarized in Supplementary Table S3. *E. coli*-specific Resfinder searches were also performed on the center for genomic epidemiology v4.1 (Bortolaia et al., 2020). SerotypeFinder v2.0 (Joensen et al., 2015) was used to perform genetic serotyping of all ETEC/STEC strains. Overall, an 80% nucleotide identity and 60% query coverage cut-off were applied. All genome assemblies were subjected to annotation using Prokka v1.14.5 (Seemann, 2014). These annotations were used in a gene-based genome-wide associations study (GWAS) using Roary v3.13.0 (Page et al., 2015) and Scoary v3.6.16 (Brynildsrud et al., 2016) along with a k-mer based approach using DBGWAS v0.5.4 (Jaillard et al., 2018). Also, known genes (e.g., rRNA-associated genes and *pmr* operons) were manually extracted to screen for potential known markers. The predictive power of associated genetic markers was calculated as previously described by Shim et al. (2019), which included the generation of confusion matrix (i.e., true positives, true negatives, false positives, false negatives) to be used in the meta v4.20–2 (Schwarzer et al., 2015) and metafor v3.8.1 (Viechtbauer, 2010) packages. The former allowed to log transform the data (sm = “PLN” in meta function) and determine Clopper-Pearson (CP) confidence intervals (method.ci = “CP” in meta function). The metafor package was used to generate forester plots using the forester function in R v4.2.1. An overview of these results is given in Supplementary Table S4 (Shim et al., 2019). Results of multiplex PCR and phenotypic resistance based on NRI-based ECOFF values were considered as “gold” standard in these calculations. To study the plasmid landscape, in-depth characterization and mobilization potential of virulence- and ARG-encoding plasmids was done using the MOB-suite v3.1.0 (Robertson and Nash, 2018). A complete overview is given in Supplementary Table S5.

## 3. Results

### 3.1. Predictive power of virotyping using whole-genome sequencing

**Figure 1:**
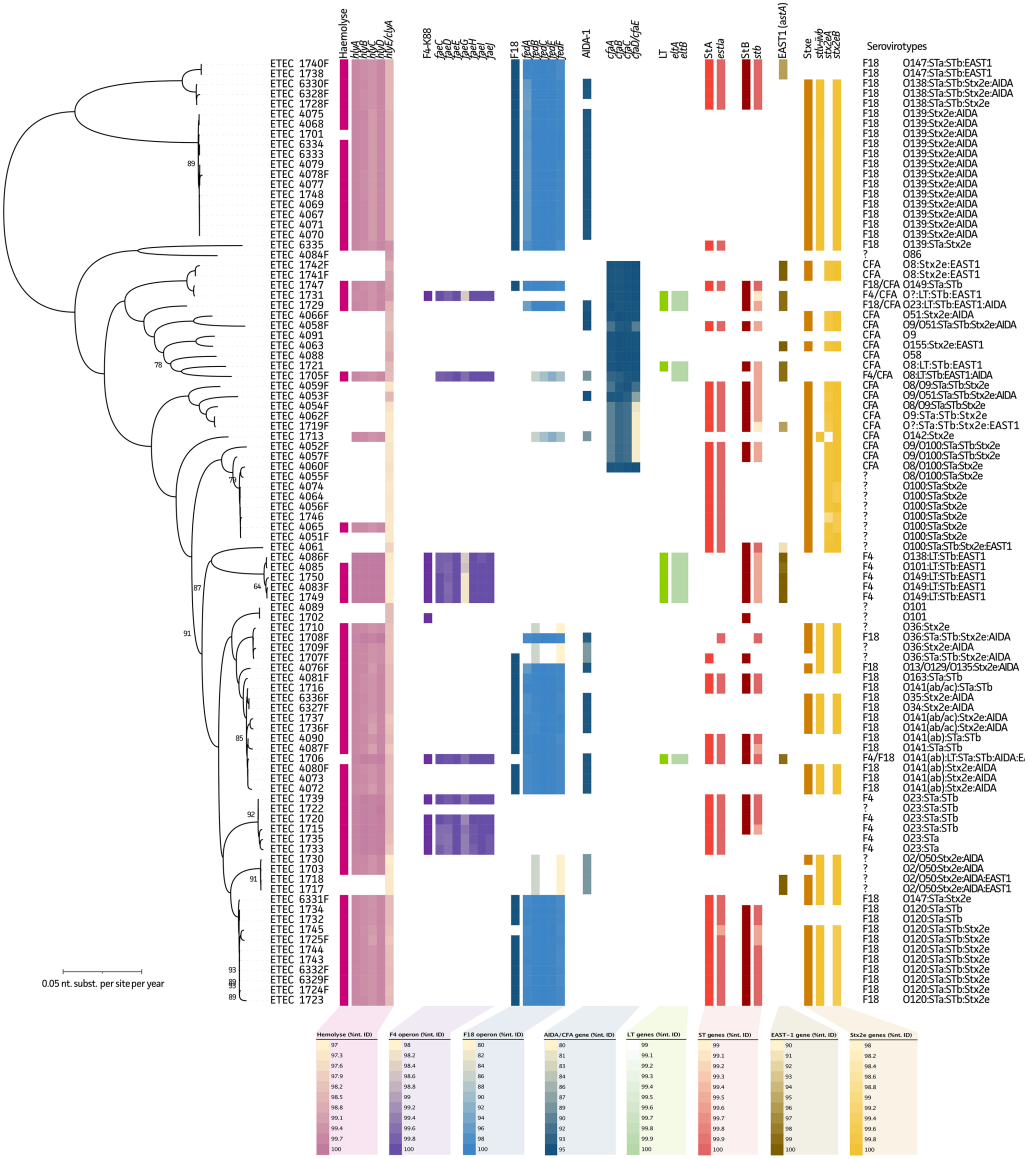
Maximum Likelihood (ML) tree of 94 ETEC/STEC strains in relation to their serovirotypes. Phylogenetic tree highlights the diversity of the studied ETEC/STEC strains and the presence of fimbrial and virulence genes. From left to right in the tree: hemolysis (as observed from blood agar plates), *hly(A-E)* genes, F4 fimbriae (multiplex PCR), fae(C-J) genes, F18 fimbriae (multiplex PCR), fed(A-F) genes, non-fimbrial AIDA gene, LT (multiplex PCR), eltA/eltB genes, STa (multiplex PCR), estIa gene, STb (multiplex PCR), stb gene, astA gene, Stx2e (multiplex PCR), and Stx2eA/Stx2eB genes. Note, two different Stx2eA variants were identified. Complete serovirotypes are given at the utmost right and legends represent nucleotide identity ranges of genes obtained from WGS data.

First, nanopore sequencing quality was assessed by determining the genome completeness and sequencing accuracy of triplicate *E. coli* ATCC 25922 genome assemblies. This showed a completeness of 99.64% and Q50 accuracy, which is in line with the available reference sequence (NZ_CP009072.1; 99.64% completeness and Q50). Thus, the current sequencing workflow allows to deliver complete and accurate genome assemblies for subsequent in-depth ETEC/STEC sequence analyses. Virotyping was initially performed using a standard multiplex PCR and associated gel electrophoresis. Alpha-hemolysis was determined based on observations on blood agar cultures. As compared to the presence of the plasmid-borne *hly(CABD)* genes from the long-read genome assemblies, 62 and 28 true positives and negatives were observed, respectively. Only for four strains showed discrepancies (false positives) between the observed phenotypes and genotypes. This resulted in a 100%/88% sensitivity/specificity (Sn/Sp) measure. A fifth chromosome-encoded *hlyE* (or *clyA*) gene was identified in all strains, which showed significant deviations in nucleotide identities in various strains (Figure 1, pink).

Like hemolysis, genome assemblies were screened for fimbrial and toxin genes to evaluate their predictive power as compared to the “gold” standard multiplex PCR data. For fimbrial adhesins F4 and F18, 12/80 and 42/48 true positives/negatives were shown, respectively. Due to the presence of one false-positive and one false-negative strain for F4, its predictive measures showed a Sn/Sp of 92%/99% (Figure 1, purple). For F18, a 100%/92% Sn/Sp was observed due to the presence of four false positives. In seven strains, diverging *fedB* and *fedF* genes were found, without the presence of other F18-associated genes (Figure 1, blue). A gene encoding the non-fimbrial protein AIDA was also identified in 37 strains, of which 34 showed the presence of *stx2e* genes. In 35 out of the 94 strains, no F4 nor F18 could be identified. Still in 17 of these strains the *cfa(ABCEF)* operon (CFA/I-like colonization factor) was present, which showed diverging nucleotide identities in some strains. In four other strains, the co-existence of the *cfa(ABCEF)* operon was seen with the F4 (*n* = 2) and F18 (*n* = 2) fimbriae (Figure 1, dark blue). For the other strains, no known (non-)fimbrial proteins were detected.

Heat-labile enterotoxin genes *eltA* and *eltB* showed a 100%/99% Sn/Sp (9/84 true positive/negatives and one false positive), whereas the heat-stable enterotoxins STa (*estIa* gene) and STb (*stb* gene) represented a 98%/98% (46/46 true positives/negatives and one false positive and negative each) and 95%/96% (42/48 true positives/negatives and two false positive and negative each) predictive power, respectively (Figure 1, green, light, and dark red, respectively). In addition, 20% of these strains showed the presence of the plasmid-encoded EAST1 (*astA* gene), which was mainly (63%) present in non-STEC strains. The gene collocated on a plasmid with the LT (*eltA* and *eltB*) and *stB* gene in ten strains (Figure 1, dark brown and see further). Finally, for the *stx2eA* and *stx2eB* genes, a Sn/Sp measure of 100%/94% (62/30 true positives/negatives and two false positives) was obtained (Figure 1, yellow). A detailed overview of the predictive measures and 95% confidence intervals can be found in Supplementary Table S4.

As summarized in Table 1, all F4 and F18 serovirotypes showed hemolytic activity. For F4 ETEC/STEC strains, five different serovirotypes were identified. Three of these showed the presence of the LT, STb, and EAST1 toxins. In the F18 fimbriated strains, up to 17 different serovirotypes were observed with ten different O serogroups (O13, O34, O35, O36, O120, O138, O139, O141, O147, and O163). In eight serovirotypes the *stA* and *stB* genes were present. While the *stx2e* genes were identified in 12 of these virotypes, EAST1 and AIDA were identified in one and eight serovirotypes, respectively. Within the CFA group, no hemolytic strains were observed. A total of ten different serovirotypes were detected, of which two showed no virulence genes (O9 and 058). The *stx2e* genes were identified in seven CFA-positive serovirotypes. In strains without known fimbriae, four out of 14 serovirotypes showed the presence of the non-fimbrial adhesin AIDA. Interestingly, only six serovirotypes showed hemolytic features and 11 showed the presence of the *stx2e* genes. Also, a wide variety of other virulence factors have been described. In the current dataset the *eaeH*, *cah*, and *tia* genes were identified, though their actual role in porcine disease is not clear yet (*data not shown*). Noteworthy, three isolates (4053, 4058, and 4066) showed the chromosomal presence of intimin, also known as AE factor Eae (*data not shown*). Two strains showed the absence of any of the studied virulence genes, which belonged to the O86 and O101 serotypes. Two double CFA/F4 and CFA/F18 strains were found, which clustered within the CFA group (Figure 1, dark blue). Finally, one double F4/F18-positive strain (1706) was identified showing an O141(ab):LT:STa:STb:AIDA:EAST1 serovirotype. Interestingly, the multiplex PCR typed this strain F4 positive, but the WGS data clustered the strain within an F18-positive cluster (Figure 1, blue).

**Table 1:** Overview of the different obtained serovirotypes.

Fimbrial adhesin	serovirotypes	Fimbrial adhesin	serovirotypes	Fimbrial adhesin	serovirotypes	Fimbrial adhesin	serovirotypes
F4	O23:STa	F18	O13/O129/O135:Stx2e:AIDA	CFA	O8:Stx2e:EAST1	Unknown	O2/O50:Stx2e:AIDA
	O23:STa:STb		O34:Stx2e:AIDA		O8:LT:STb:EAST1		O2/O50:Stx2e:AIDA:EAST1
	O101:LT:STb:EAST1		O35:Stx2e:AIDA		O8/O9:STa:STb:Stx2e		O8/O100:STa:Stx2e
	O138:LT:STb:EAST1		O36:STa:STb:Stx2e:AIDA		O8/O100:STa:Stx2e		O9:STa:STb:Stx2e
	O149:LT:STb:EAST1		O120:STa:STb		O9/O51:STa:STb:Stx2e:AIDA		O23:STa:STb
			O120:STa:STb:Stx2e		O9/O100:STa:STb:Stx2e		O36:Stx2e	O138:STa:STb:Stx2e	O51:Stx2e:AIDA	O36:Stx2e:AIDA	O138:STa:STb:Stx2e:AIDA	O155:Stx2e:EAST1	O36:STa:STb:Stx2e:AIDA	O139:Stx2e:AIDA	O9	O100:STa:Stx2e*	O139:STa:Stx2e	O58	O100:STa:STb:Stx2e:EAST1
F18/CFA	O23:LT:STb:EAST1:AIDA		O141:STa:STb		O142:Stx2e	
	O149:STa:STb		O141(ab):Stx2e:AIDA		O?:STa:STb:Stx2e:EAST1	
			O141(ab/ac):Stx2e:AIDA		O86	
F4/CFA	O8:LT:STb:EAST1:AIDA		O141(ab/ac):STa:STb		O101	
	O?:LT:STb:EAST1		O147:STa:Stx2e			
			O147:STa:STb:EAST1			
F4/F18	O141(ab):LT:STa:STb:AIDA:EAST1		O163:STa:STb			

Serovirotypes are shown based on their fimbrial classification, being F4 or F18 positive. If not identified strains were placed into the category ‘unknown’. Serotypes are indicated as O antigens and all observed virulence genes. If hemolysis was observed, serovirotypes were highlighted in red. Only one (out of 6) strain within the O100:STa:Stx2e serovirotype (*) showed the presence of hemolytic genes.

### 3.2. Antimicrobial resistance and genetic hallmarks

#### 3.2.1. Beta-lactams, cephalosporins, and polymyxin E

**Figure 2:**
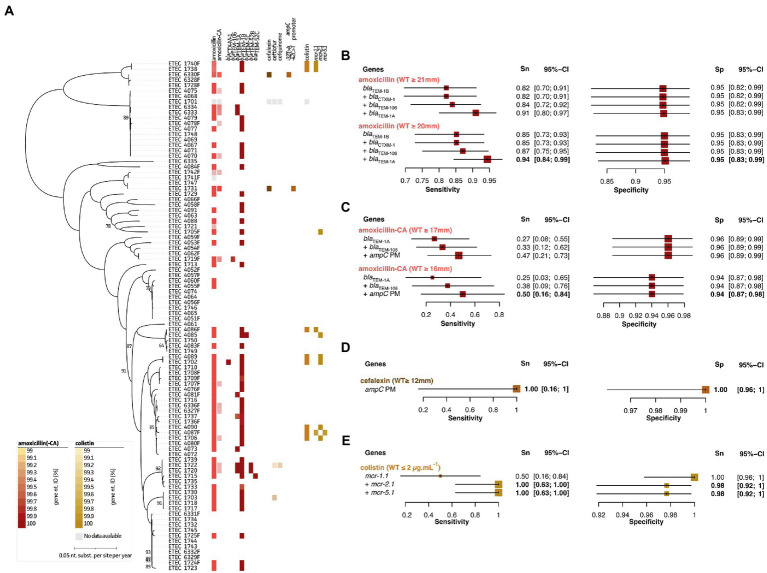
Association analyses for phenotypic and genotypic antimicrobial resistance of beta lactams, cephalosporins, and colistin. **(A)** Maximum Likelihood (ML) tree showing strain relatedness and distribution of resistance phenotypes and genetic markers. Resistance phenotypes are shown based on NRI ECOFF values in which white and colored boxes represent WT and non-WT population, respectively. From left to right; amoxicillin phenotypes, amoxicillin + clavulanic acid (CA) phenotypes, *bla* genes, cefalexin phenotypes, ceftiofur phenotypes, cefquinome phenotypes, *ampC* promotor mutations, colistin phenotypes (E-test), and *mcr* genes. Color grades for genes indicate nucleotide identities (%) as indicated in the legends at the left bottom; **(B–E)** Forest plots of predictive measures for selected genetic markers and ECOFF diameter cut-offs for the observed phenotypes (disc diffusion diameters (mm) or mg.mL-1 for colistin only). Forest plots show Sensitivity (Sn) and Specificity (Sp) measures and their 95% confidence intervals (CI). Whenever a “+” is indicated, the genetic marker was added to generate the confusion matrix for the analysis.

Figure 2A and Supplementary Figure S1 highlight the observed zone of inhibition diameters (mm) and MIC distribution (for colistin only) for all tested antimicrobials belonging to the category of beta-lactams (amoxicillin and amoxicillin + clavulanic acid (CA)), cephalosporins (cefalexin, ceftiofur, and cefquinome), and polymyxins (colistin). For amoxicillin, majority (57%) of the strains belonged to the non-WT population based on the NRI ECOFF, which could be addressed by three plasmid-encoded beta-lactamases (Supplementary Figure S1). The presence of either one or combinations of *bla*_TEM-1A_, *bla*_TEM-1B_, and/or *bla*_TEM-106_ showed a 91%/95% Sn/Sp (50/37 true positives/negatives and 2/5 false positives/negatives) for our strains. Two of the false negatives (strains 1721 and 1742F) showed diameters of 20 mm, which was just below the set ECOFF (≥ 21 mm). Adjusting the ECOFF value to ≥20 mm, improved the Sn/Sp measures to 94%/95%. The *bla*_TEM-1B_ gene was the most abundant in 49 of all strains. Also, *bla*_CTXM-1_ (*n* = 1), *bla*_TEM-52A_ (*n* = 2), and *bla*_TEM-52B_ (*n* = 1) were identified, but did never occur alone (Figure 2B, red). In two strains (1720 and 1722), *bla*_TEM-1A_, *bla*_TEM-1B_, and *bla*_TEM-52B_ were present on three independent plasmids. A total of 14 strains were categorized to the non-WT population of amoxicillin + CA of which *bla*_TEM-1A_ and *bla*_TEM-106_ were identified in two and one strain, respectively. Interestingly, these strains showed diameters of 11 and 12 mm, respectively. Still, the *bla*_TEM-1A_ gene was also identified in five other strains with diameters ranging between 16 and 18 mm. Seven other strains with diameters of 16 mm, just below the set ECOFF (≥ 17 mm), only harbored the *bla*_TEM-1B_ gene. The two remaining strains showed the lowest diameters (6 and 8 mm, respectively) for amoxycillin + CA, which did not show the presence of any beta-lactamases. Instead, they harbored point mutations (−32 T > A or −42C > T) within the *ampC* promotor region. For this genetic marker, in combination with *bla*_TEM-1A_ and *bla*_TEM-106_ at an ECOFF of ≥17 mm (NRI), the Sn/Sp only showed 47%/96% (7/76 true positives/negatives and 3/8 false positives/negatives). Here again, changing the ECOFF to ≥16 mm improved the Sn to 50%, but lowered the Sp to 94% (4/81 true positives/negatives and 5/4 false positives/negatives) (Figure 2C; red). Mutations in the *ampC* promoter region also coincided with acquired resistance in both strains against the cephalosporine cefalexin, suggesting a cross-resistance mechanism (Figure 2D, dark brown). Based on the ECOFF (NRI) value (≥12 mm), strain 4089 still belonged to the WT population. This resulted in a 100%/100% Sn/Sp measure (2/92 true positives/negatives). Indeed, the ECOFF clearly separates the population into a WT and non-WT (resistant) population (Supplementary Figure S1). For the other tested cephalosporins (cefquinome and ceftiofur) two and one strains were categorized to the non-WT population (Supplementary Figure S1). Due to the low number of samples in the non-WT population, no GWAS-based analyses could be performed. Noteworthy, the diameter difference between the cut-off and strain with lowest diameter was only 3 mm. Finally, colistin resistance was mediated by three different *mcr* genes, including *mcr-1.1*, *mcr-2.1*, and *mcr-5.1*. The *mcr-1.1* gene was present in four out of eight resistant strains, the *mcr-2.1* and *mcr-5.1* in six and one strain, respectively. While the sensitivity was 100%, a slight reduction in specificity (100 to 98%) was observed, due to the presence of *mcr* genes in two strains that did not show phenotypic resistance against colistin when considering the ECOFF value (≤2 μg.mL^−1^). (Figure 2E, light brown). All genomes were also screened for amino acid mutations in proteins involved in the regulation of colistin-associated resistance mechanisms (i.e., pmrA, pmrB, phoP, phoQ, mgrB, pmrHFIJKLM), some previously studied mutations were identified (e.g., the V161G in pmrB; *data not shown*). Though, none could be additionally associated with the observed resistance phenotypes. An overview of the predictive values and 95% confidence intervals can be found in Supplementary Table S4.

#### 3.2.2. Aminoglycosides and amphenicols

**Figure 3:**
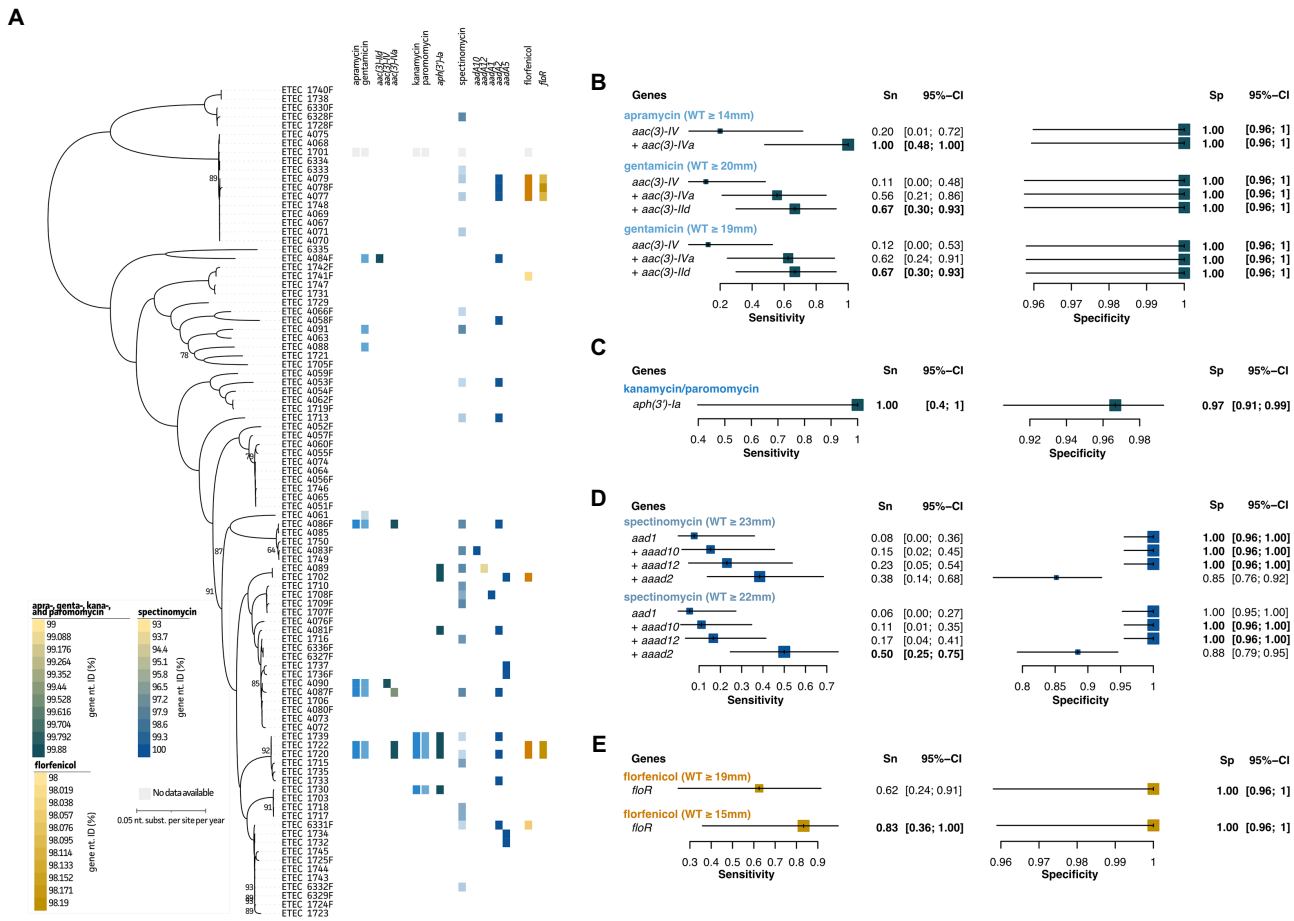
Association analyses for phenotypic and genotypic antimicrobial resistance of aminoglycosides and florfenicol. **(A)** Maximum Likelihood (ML) tree showing strain relatedness and distribution of resistance phenotypes and genetic markers. Resistance phenotypes are shown based on NRI ECOFF values in which white and colored boxes represent WT and non-WT population, respectively. From left to right; apramycin and gentamicin phenotypes, *aac* genes, kanamycin and paromomycin phenotypes, *aph* genes, spectinomycin phenotypes, *aad* genes, florfenicol phenotypes, and *floR* gene. Color grades for genes indicate nucleotide identities (%) as indicated in the legends at the left bottom. **(B–E)** Forest plots of predictive measures for selected genetic markers and ECOFF diameter cut-offs for the observed phenotypes (disc diffusion diameters (mm) or μg.mL^−1^ for colistin only). Forest plots show Sensitivity (Sn) and Specificity (Sp) measures and their 95% confidence intervals (CI). Whenever a “+” is indicated, the genetic marker was added to generate the confusion matrix for the analysis.

As shown in Figure 3A and Supplementary Figure S1, only a few strains showed acquired resistance to the studied aminoglycosides. These included apramycin (*n* = 5), gentamicin (*n* = 9), kanamycin (*n* = 4), paromomycin (*n* = 4), and spectinomycin (*n* = 24). The antimicrobial family of aminoglycosides was split up in three categories. For apramycin and gentamycin, clear associations with aminoglycoside 3-N-acetyltransferase genes *aac(3)-IId*, *aac(3)-IV*, and *aac(3)-IVa* could be made. These genes allowed to predict five out of nine strains with resistance (Figure 3B, light blue). Interestingly, while *aac(3)-IId* seemed to confer resistance toward gentamicin only, the other two genes showed cross-resistance toward both apramycin and gentamicin. Using the *aac(3)-IV* and *aac(3)-IVa* genes as predictive measures for apramycin, allowed to give a 100%/100% Sn/Sp. Adding the *aac(3)-IId* genetic marker for gentamicin AST prediction, showed a 67%/100% Sn/Sp. Lowering the NRI-based ECOFF to 19 mm, did not result in changes in the predictive measures here (Figure 3B, light blue). Next, kanamycin and paromomycin were grouped together for which the aminoglycoside-phosphotransferase *aph(3*′*)-Ia* gene was identified as best predictor (100%/97% Sn/Sp; Figure 3C, light blue). Even though for spectinomycin different reference databases, *E. coli*-specific Resfinder, gene-, and k-mer-based GWAS identified various adenylyltransferase (*aadA*) genes, no clear associations could be made. The *aadA1*, *aadA2*, *aadA5*, *aadA10*, and *aadA12* genes were identified in the dataset, but only *aadA1* (*n* = 1), *aadA10* (*n* = 1), and *aadA12* (*n* = 1) showed apparent correlation with the observed phenotypes. Even though the *aadA2* gene was present in 14 genomes, only nine strains showed a spectinomycin resistance phenotype (Figure 3D, dark blue). Due to the antibiotic mechanism of aminoglycosides, 16S rRNA gene sequences were screened for known point mutations (*data not shown*). Still, no correlations with the observed phenotypes could be made. Nevertheless, a non-WT (resistant) population was observed for spectinomycin (Supplementary Figure S1). Using the *aad1*, *aad2*, *aad10*, and *aad12* genes and NRI-based ECOFF (WT ≥ 23 mm), Sn/Sp measures of 38%/85% were obtained. Lowering this to ≥22 mm, slightly increased both Sn and Sp to 50 and 88%, respectively (Figure 3D; dark blue). Finally, predictive measures could be obtained for acquired resistance toward florfenicol, using the the presence of the plasmid-encoded *floR* gene (Figure 3E, light brown). This gene showed an 62%/100% Sn/Sp at the NRI-based ECOFF (WT ≥ 19 mm). Though, based on the visual (apparent) split of the non-WT and WT population, a lowered cut-off (WT ≥ 15 mm) resulted in an improved Sn of 83%, maintaining a 100% Sp (Figure 3E, light brown). A detailed overview of the predictive values and 95% confidence intervals can be found in Supplementary Table S4.

#### 3.2.3. Tetracyclines, fluoroquinolones, and trimethoprim/sulfamethoxazole

**Figure 4:**
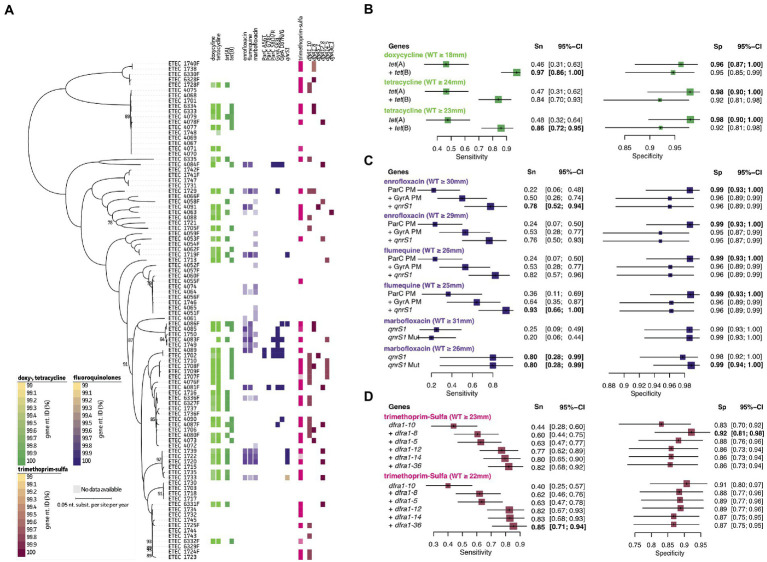
Association analyses for phenotypic and genotypic antimicrobial resistance of tetracyclines, fluoroquinolones, and trimethoprim-sulfa. **(A)** Maximum Likelihood (ML) tree showing strain relatedness and distribution of resistance phenotypes and genetic markers. Resistance phenotypes are shown based on NRI ECOFF values in which white and colored boxes represent WT and non-WT population, respectively. From left to right; doxycycline and tetracycline phenotypes, *tet* genes, enrofloxacin, flumequine, and marbofloxacin phenotypes, *ParC* protein mutations, *GyrA* protein mutations, *qnrs1* gene, trimethoprim-sulfa phenotypes, and *dfrA* genes. Color grades for genes indicate nucleotide identities (%) as indicated in the legends at the left bottom. **(B–D)** Forest plots of predictive measures for selected genetic markers and ECOFF diameter cut-offs for the observed phenotypes (disc diffusion diameters (mm) or μg.mL^−1^ for colistin only). Forest plots show Sensitivity (Sn) and Specificity (Sp) measures and their 95% confidence intervals (CI). Whenever a “+” is indicated, the genetic marker was added to generate the confusion matrix for the analysis.

Again, disc zone diameter distributions (mm) are represented in Figure 4A and Supplementary Figure S1. For the tetracycline doxycycline associations could be made using the *tet*(A) and *tet*(B) genes (97%/95% Sn/Sp). For doxycycline associations could be obtained after changing the NRI-based ECOFF value (WT ≥ 24 mm to ≥23 mm), the Sn improved from 84 to 86%, while maintaining an Sp of 92% (Figure 4B; green). A total of 37 true positive samples were detected of which three showed the presence of both genes. All other samples had either one of both genes present. Two different mechanisms were identified to contribute to acquired resistance in fluoroquinolones. On the one hand, point mutations were identified within the GyrA (A56T, G78C, and S80I/R) and/or ParC (S83L and 87 N/G) proteins of 12 strains (Figure 4C, purple). For most strains with lowered disc inhibition diameters (i.e., non-WT population), combined mutations in both GyrA and ParC were present. Only for some strains a single point mutation was identified. While strain 1729 and 4085 showed a single mutation (S83L in GyrA), the strains were still classified to the flumequine and enrofloxacin/flumequine non-WT population, respectively (Supplementary Figure S1). On the contrary, strain 4087, 4090, 1749, and 1750 also harbored a single point mutation (S83L or D87N/G in GyrA), though they were not considered resistant (Figure 4A, purple). For six strains, the quinolone resistance gene *qrnS1* was identified, which seemed to confer resistance toward all three tested fluoroquinolones in four out of five strains. Interestingly, mutations in the *qrnS1* gene seemed to result in lowered acquired resistance. This was exemplified for strain 1733 (lowered zone diameters for all fluoroquinolones; Figure 4A). For enrofloxacin, previously described genetic markers showed predictive measures of 78%/96% Sn/Sp at the NRI-based ECOFF (WT ≥ 30 mm; Figure 4C, purple). For both flumequine and marbofloxacin, lowering the NRI-based ECOFF value to WT ≥ 25 mm (instead of WT ≥ 26 mm) and WT ≥ 26 mm (instead of WT ≥ 31 mm), allowed to improve the predictive measures. A final 93%/96% Sn/Sp was obtained for flumequine when using the three genetic markers. For marbofloxacin, only the *qnrS1* marker was used, showing increased Sp (98 to 99%) when considering the lowered resistance when significant mutations are identified in the *qnrS1* gene (99.24% nt. ID; Figure 4A). A total of 44 strains belonged to the trimethoprim-sulfamethoxazole non-WT population (Figure 4D, pink). An association was observed for various dihydrofolate reductase (*dfrA*) genes. A combination of *dfrA1*, *dfrA5*, *dfrA12*, *dfrA14*, and *dfrA36* allowed to predict phenotypic resistance up to 82%/86% Sn/Sp. Also here, lowering the NRI-based ECOFF to WT ≥ 22 mm allowed to increase the Sn to 85%, maintaining a 86% Sp. A detailed overview of the predictive values and 95% confidence intervals can be found in Supplementary Table S4.

### 3.3. Characterization and potential dissemination of plasmid-borne virulence and resistance

To perform in-depth characterization of plasmids, only complete circular genomes and plasmids (Trycycler) were included in this analysis. The MOB-suite was used to characterize each plasmid and assess potential dissemination. A complete overview of this analysis can be found in Supplementary Table S5. Overall, virulence and resistance genes were found to be located on different plasmids. Only some exceptions were observed. As summarized in Table 2, for ETEC strain 1715 and 1720, a *bla*_TEM-1B_ gene collocated on the plasmid carrying the *fae(CDEFGHIJ)* operon. In strain 4085 the *astA* and *stb* genes were found on the same plasmid carrying *bla*_TEM-1B_. Indeed, these three strains showed resistance toward amoxicillin (Figure 3A). The *aadA5* and *dfrA17* gene were found on a *hly(CABD)* and *fed(ABCEF)* plasmid in strain 1737, although these genes were not associated with resistance in our dataset (Figures 4A, D). Finally, in strain 4085 the *hly(CABD)* operon was located next to genes conferring resistance toward beta-lactam (*bla*_TEM-1C_) and aminoglycosides (*aph(6)-Id, aph(4)-Ia,* and *aph(3*″*)-Ib*) among others.

**Table 2:** In-depth characterization of virulence-encoding plasmids.

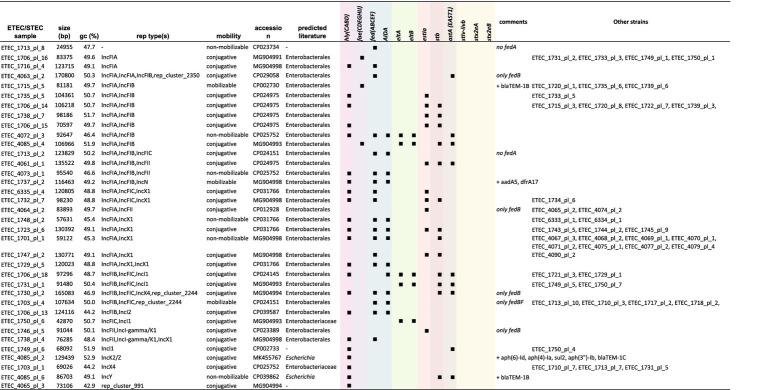

Summarizing table of all plasmid types identified in complete and circular (Trycycler) genomes, highlighting plasmid size (bp), GC content (%), replicon (rep) type, mobility potential based on MOB-suite results, and the closest known plasmid sequence accession. Host range was predicted in the MOB-suite and is represented as supported by literature. Virulence markers which were the focus of this manuscript were identified and colored in compliance to Figure 1. A complete output can be found in Supplementary Table 5.

Virulence-encoding plasmids were categorized in a minimum of 36 different types, of which 27 were classified as conjugative. Three others were considered mobilizable and six non-mobilizable. The plasmid host range was “limited” to the Enterobacterales for all plasmids. Interestingly, two plasmids showed the lack of the *fedA* gene but the presence of all other F18 operon genes. On the contrary, four plasmids showed the presence of only the *fedB* gene. One of these also showed the presence of the *fedF* gene. This was also reflected in Figure 1. In 16 plasmids, the *hly(CABD)* operon was identified next to the *fed(ABCEF)* operon. Only three different F4 type plasmids were identified, of which two did not show the presence of other virulence factors and one harboring *eltA*, *eltB*, *stb*, and *astA* genes. Also, 15 plasmids were identified to not harbor any of the fimbrial genes. While some only harbored the *hly(CABD)* operon, the *eltA*, *eltB*, *estIa*, *stb*, and *astA* genes were identified in three, three, seven, seven, and five plasmids, respectively. Most of these plasmids (28 out of 36) were classified as multi-replicon. The majority harbored either one of both IncF1A (*n* = 20) and IncF1B (*n* = 17) replicons, eventually together with an IncX1 (*n* = 8), IncF1C (*n* = 7), IncFII (*n* = 5), IncI1 (*n* = 3), IncI-gamma/K1 (*n* = 2), Incl2 (*n* = 1), or IncN (*n* = 1) replicon on the same plasmid.

A minimum of 50 different plasmids were identified to carry AMR genes. Only seven plasmids were classified to be non-mobilizable and 41 were considered conjugative. As summarized in Table 3, a wider host range of conjugation and mobilization potential was observed for the AMR plasmids. These AMR plasmids showed mobilization potential from narrow host ranges, such as within *Escherichia* species (e.g., ETEC 4085 plasmid 2 with five AMR genes) to Enterobacterales (e.g., ETEC 1702 plasmid 2 with seven AMR genes) and even broad host ranges, such as Proteobacteria-wide dissemination (e.g., ETEC 1729 plasmid 2 with eight AMR genes). While a multitude of other plasmids without AMR genes were identified, one conjugative plasmid was found to harbor 12 AMR genes in ETEC strain 4077. This plasmid had IncFIA rep, MOBH relaxase, MPF-F mpf types (Table 2; Supplementary Table S5). Resistance genes toward beta-lactams (*bla*_TEM-1B_), aminoglycosides (*ant(3*″*)-Ia, aadA2, aph(6)-Id, and aph(3*″*)-Ib*), florfenicol (*floR*), and tetracycline (*tet*(B)) were found among others. The plasmid was identified in three strains (ETEC 4077, 4078F, and 4079), which showed phenotypic resistance toward amoxycillin, doxycycline, tetracycline, and florfenicol (Figures 2G, 4G). While virulence-associated plasmids were mostly multi-replicon, only 16 (out of 50) AMR plasmids showed the presence of more than one replicon type. The IncI-gamma/K1 replicon dominated as it was observed in 15 plasmids, followed by IncF1A (*n* = 8) and IncF1B (*n* = 5), eventually together with a IncF1C (*n* = 5), IncQ1 (*n* = 5), rep_cluster_1088 (*n* = 4), IncHI2A (*n* = 3), IncN (*n* = 2), IncX1 (*n* = 2), IncX3 (*n* = 2), IncI-gamma/K1 (*n* = 2), or IncFII (*n* = 1) replicon on the same plasmid.

**Table 3:** In-depth characterization of AMR gene-encoding plasmids.

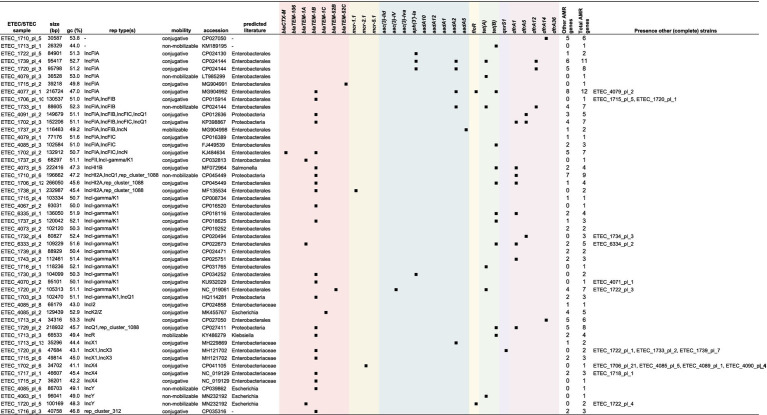

Summarizing table of all plasmid types identified in complete and circular (Trycycler) genomes, highlighting plasmid size (bp), GC content (%), replicon (rep) type, mobility potential based on MOB-suite results, and the closest known plasmid sequence accession. Host range was predicted in the MOB-suite and is represented as supported by literature. Antimicrobial resistance genes were identified and colored in compliance to previous Figures 2–4. A complete overview can be found in Supplementary Table 5.

## 4. Discussion

In the current study, we applied long-read nanopore sequencing on 94 porcine ETEC/STEC field isolates to determine the predictive power of existing and future sequencing-based diagnostic workflows for combined virulence and genetic AST. Virulence and AMR-associated genes obtained from WGS data were evaluated against data from a virulence multiplex PCR and phenotypic disc diffusion tests, respectively. In addition, our data allowed to assess serovirotypes, along with in-depth characterization of plasmids since complete circular assemblies were obtained from long-read data.

Predictive estimates for alpha-hemolysis, fimbriae (F4 and F18), and enterotoxins (LT, STa, STb, and Stx2e) were high. A sensitivity of 100% was observed for hemolysis (*hly(CABD)*), F18 (*fed(ABCEF)*), LT (*eltA* and *eltB*), and Stx2e (*Stx2eA* and *Stx2eB* genes). The lowest specificity measure (88%) was observed for the hemolysis genes (*hly(CABD)*), which might be explained by the fact that alpha-hemolysis is not always easy to interpret on blood agar plates. It is highly dependent on the metabolic state of the strain, which is impacted by repeated passaging and extended incubation times (Short and Kurtz, 1971; Schwidder et al., 2019). Different STEC strains were shown to require adjusted blood agar plates to show hemolytic activity (Lehmacher et al., 1998; Lorenz et al., 2013). For the tested fimbriae and enterotoxins, specificity ranged from 92.3% up to 98.9%. These high values are not surprising as both methods rely on genetics and not on phenotypes (as compared to hemolysis and AST). While multiplex PCR assays were shown to be as sensitive as phenotypic ELISA or dot blotting, they are able to detect actively expressed and silenced genes (Rodas et al., 2009; Botkin et al., 2012). This is considered an advantage as this allows to perform a preliminary screening at the highest specificity and sensitivity (Sjöling et al., 2007). Moreover, negative phenotypic characterization does not imply that genes are not expressed during infection. Actual expression is highly dependent on storage, cultivation, growth medium, and regulation at various levels (Sjöling et al., 2007; Fairbrother and Nadeau, 2019). Hence, sequencing represents a perfect tool to address virotyping of ETEC/STEC strains.

Since the current study did not focus on delivering epidemiological data, no conclusions on the prevalence of serovirotypes will be made. Nevertheless, our data allowed to address the wide variety of serovirotypes. Identification of double positive strains (e.g., F18/CFA, F4/CFA, and F4/F18) might be because of mixed populations (Francis, 2002; de Lagarde et al., 2021) or hybrid ETEC/STEC strains as previously reported in Sweden (Bai et al., 2019). Overall, up to 17 different serovirotypes were identified, including the common O139:Stx2e:AIDA (Fairbrother and Nadeau, 2019). Most common serogroups were also present in our dataset as observed in recent studies from Australia, Canada, and South-Korea (Abraham et al., 2012; de Lagarde et al., 2021; Byun et al., 2022; Do et al., 2022). For the F18 group, all strains showed alpha-hemolytic features as previously reported (Fairbrother and Nadeau, 2019). The non-fimbrial adhesin AIDA was also identified in a wide variety of serovirotypes, which seemed not to be limited to the STb or STb:EAST1 virotypes as previously reported (Mainil et al., 2002; Fairbrother and Nadeau, 2019). Our dataset confirms the controversial role of EAST1 as it was never found alone in any of the virotypes. It was suggested that EAST1 on its own is unable to result in disease phenotypes, but requires the LT enterotoxin (Dubreuil et al., 2016). We observed LT and EAST1 to be co-occurring in F4 virotypes, though other combinations with EAST1 were observed in F18 and virotypes with unknown colonization factors. To the authors knowledge, this is one of the few reports on pig ETEC/STEC strains that includes the *cfa(ABCEF)* operon in pathotyping. While most studies perform targeted multiplex PCRs to characterize pathogenic *E. coli* strains, WGS facilitates the identification of a wider variety of adhesins and enterotoxins (e.g., *eae* and *cfa(ABCEF)*), without prior PCR selection (Botteldoorn et al., 2003; Casey and Bosworth, 2009; Abraham et al., 2012; Byun et al., 2022; Do et al., 2022).

So far, sequencing has not been encouraged in AST since time-consuming phenotypic alternatives are still considered the “gold” standard. Nevertheless, implementing sequencing-based (meta)genomics could speed up diagnostics of enteric colibacillosis significantly by delivering strain-level identification, pathotyping, and genetic AST. Our data showed promising results for most of the relevant antimicrobial drugs. Over the last years, a multitude of studies reported a variety of resistance genes without proper associations with phenotypes (Eyre et al., 2017; Nguyen et al., 2018). Nevertheless, ARG database searches often result in extensive lists of genes, which do not necessarily contribute to phenotypic resistance. By applying combined searches of databases (e.g., abricate and Resfinder) in combination with GWAS tools (e.g., gene- and k-mer based) a more comprehensive output can be generated (Jaillard et al., 2018; Farhat et al., 2019; Bokma et al., 2021; Weber et al., 2021; Cardenas-Alvarez et al., 2022). Here, good predictive power was shown for 13 different antimicrobial drugs for which a non-WT population (ECOFF) was determined based on the NRI method for their AMR phenotypes. Interestingly, a combination of 25 ARGs and seven point mutations allowed genetic AST of ETEC/STEC strains. Sensitivity measures ranged from 50% (spectinomycin: *aadA* genes), to 67% (gentamicin: *aac* genes), to 85% (trimethoprim-sulfa; *dfrA* genes) 97% (doxycycline: *tet* genes) up to 100% (kanamycin/paromomycin: *aph* genes; colistin: *mrc* genes). The lowest predictive measures were obtained for spectinomycin (38%/85% Sn/Sp) for which various adenylyl transferase genes were used as markers. Interestingly, this could be further improved by changing the NRI-based ECOFF value (50%/88% Sn/Sp). This was the case in many of the tested antimicrobials (Supplementary Table S4). Even though standard deviations for all tested antimicrobials were acceptable as instructed in the NRI analyses, increasing the number of isolates could improve the resolution and assessment of ECOFF values further (Kronvall, 2010). Still, our genetic analyses could support in dividing the bacterial population more accurately in a WT and non-WT population. While 16S rRNA point mutations are also known to contribute to resistance, none were identified at or in the vicinity of the spectinomycin binding pocket (Johanson and Hughes, 1995). Other mechanisms might play a role here as methylation events have also been shown to be important in aminoglycoside resistance (Lioy et al., 2014). Interestingly, predictive measures for amoxicillin-CA could be elevated further by the implementation of point mutations in the *ampC* promoter. These mutations also conferred resistance to cefalexin, a mechanism that has been described to be important in resistance. These mutations result in hyperexpression of genes upstream of the *ampC* promoter region (Yu et al., 2009) and has been shown to confer resistance to beta-lactams and cephalosporins (Tuomanen et al., 1991; Bradford et al., 1999; Caroff et al., 1999; Liebana et al., 2004). For colistin, ECOFF and clinical breakpoints should be interpreted with care as previous reports showed the presence of *mcr* genes in isolates with MIC values below the ECOFF cut-off (≤ 2 μg.mL^−1^), even in isolates with MIC values ≤0.25 μg.mL^−1^ (Pogue et al., 2020). This could be the case for the two false-negative samples, which is an additional advantage for the use of NGS in bacterial AST. Even though point mutations in regulators associated with colistin resistance mechanisms (e.g., pmrA, pmrB, phoP, phoQ, mgrB, and prmHFIJKLML) were shown to be important, colistin resistance in our dataset seemed to be majorly mediated through the presence of *mcr* genes (Gogry et al., 2021). Lowered predictive values can generally be explained by the limited number of strains belonging to the non-WT population (colistin, *n* = 8; gentamicin, *n* = 9; florfenicol, *n* = 6; apramycin, *n* = 5; paromomycin, *n* = 4; cefalexin, *n* = 2; and kanamycin, *n* = 4), which represents a limitation of current study. Still, our predictive measures for amoxicillin (94%/95%), colistin (100%/98%), apramycin (100%/100%), gentamicin (67%/100%), kanamycin/paromomycin (100%/97%), florfenicol (83%/100%), doxycycline (97%/95%), tetracycline (86%/92.%), enrofloxacin (76%/95%), flumequine (93%/96%), marbofloxacin (80%/99%), and trimethoprim-sulfa (85%/87%) are highly satisfactory. The available literature showed comparable genetic associations in *E. coli* and other bacterial species (e.g., *Pseudomonas aeruginosa*) (Zankari et al., 2012; Wohlwend et al., 2015; Nageeb and Hetta, 2022; Olorunleke et al., 2022; Peng et al., 2022). These predictive measures were higher than when only ARG databases, such as the comprehensive AMR database (CARD) or Resfinder, were used (Mahfouz et al., 2020). They were also better than reported genetic predictions for *Klebsiella pneumonia* and *Neisseria gonorrhoeae* (Eyre et al., 2017; Nguyen et al., 2018). Important to note is the species-specific nature of current hallmarks as different species might harbor other mechanisms of resistance (Bortolaia et al., 2020; Nageeb and Hetta, 2022). Genetic AST harbors an interesting future as it has been shown to aid in rapid identification and genetic AST in a preterm point-of-care-setting, but also the importance of pathogen and AMR surveillance was put forward. Nevertheless, improvements in both sequencing technologies (e.g., further increase in ONT raw read accuracy to use single read instead of consensus sequence information) and bioinformatic software were stipulated to be important for its future broader implementation (Leggett et al., 2020; Li et al., 2022). Hence, the current gene marker dataset provides a solid base for sequencing-based diagnostics to address genetic AST of ETEC/STEC. This would allow to simultaneously identify, type (*cf.* virulence factors and serotypes), and perform AST in a quick and cost-efficient manner. Furthermore, advances in bioinformatics tools and the implementation of artificial intelligence (AI) algorithms are promising to increase the predictive power even further. These will contribute to its wider application in routine diagnostic laboratories (Drouin et al., 2019; Van Camp et al., 2020). Of note, current study assumed that mPCR and phenotypic disc diffusion are “gold” standard tests in the assessment of genetic validity. This is a limitation of current study as neither of both techniques can be considered as “gold” standard since their Sn/Sp are not 100%/100% Sn/Sp (Casey and Bosworth, 2009). The question remains if this is even possible within a clinical set-up. Using a third method (e.g., protein-based detection *via* ELISA) would allow to perform a more general and unbiased prediction of predictive measures using a Bayesian latent class model (BLCM) approach (Bokma et al., 2020, 2021b). This would also deliver extra functional characterization of the identified genetic markers, which would be of great interest to further characterize and understand the observed discrepancies. Though this was not the scope of current manuscript. Still, performing functional studies will be an important part in the future implementation of sequencing-based diagnostics as it will contribute to the proper biological understanding of identified mechanisms and the curation of ARG databases.

In-depth characterization of virulence- and AMR gene-encoding plasmids was performed on plasmids obtained from complete circular genomes (Trycycler) as these plasmids underwent proper circularization. This is an important consideration when addressing multi-replicon plasmids as PCR-based and short-read approaches lack sufficient resolution on this type of plasmids (Rozwandowicz et al., 2018). Importantly, plasmid sequences from native DNA can only be recovered confidently when applying a rapid transposon-based and not ligation-based nanopore library preparation (Wick et al., 2021b). Similar to the presence of various multi-replicon plasmids in our porcine ETEC/STEC strains, a recent report on clinical and food-borne pathogenic *E. coli* strains described a wide variety of replicons and host ranges (Balbuena-Alonso et al., 2022). Most virulence-carrying plasmids had an IncF replicon as reported (Balbuena-Alonso et al., 2022). Also in bovine ETEC strains, a wide variety of multi-replicon IncF-base plasmids were identified (Mainil et al., 1992). Even though the high diversity of conjugative plasmids is worrying, horizontal transmission of MGEs is often blocked due to the presence of existing incompatible plasmids in the bacterial recipient (Dimitriu, 2022). This mechanism is also referred to as entry exclusion which results in inefficient conjugation during consecutive conjugation rounds (Garcillán-Barcia and de la Cruz, 2008). Having multiple replicons within a plasmid can circumvent this as it provides maintenance of conjugation regions contributing to a broader host range and stable replication (Pilla and Tang, 2018; Qu et al., 2019). Interestingly, IncF-IA/IncF-IB only function in enteric bacteria (Kline, 1985). The IncF group plasmids are thus typically found in the family of Enterobacteriaceae and most widely described in human and animal sources (Rozwandowicz et al., 2018). Also, non-virulence or ARG-carrying plasmids often belong to the IncF group. Hence, it is thought these plasmids have been circulating in the family upon acquiring ETEC-associated virulence factors (Bergquist et al., 1986; Chaslus-Dancla et al., 1991).

The spectrum of different replicons was wider for plasmids carrying ARGs. The IncF plasmids have also been associated with the spread of ARGs, more specifically the extended-spectrum beta-lactams (e.g., *bla*_CTX-M_) (Coque et al., 2008; Marcadé et al., 2009). Indeed, also in our dataset, a plasmid carrying a *bla*_CTX-M_ gene was presented on a multi-replicon IncF-IA/IncF-IC/lncN plasmid. This combination is not surprising as IncN plasmids are known to colocalize with IncF plasmids. In addition, IncN plasmids are important players in the dissemination of *bla*_CTX-M_ throughout Europe as they are broad host plasmids (Rozwandowicz et al., 2018). Interestingly, the quinolone resistance gene, *qnrS1*, was first identified on a IncN replicon plasmid in pigs in Europe (Szmolka et al., 2011). In our ETEC/STEC strains, the *qnrS1* gene was identified on a IncX1/IncX3 multi-replicon plasmid in some strains. Even though beta-lactams are mostly associated with IncF group plasmids, our dataset showed a wide distribution of beta-lactams in various plasmids belonging to the IncH, IncI, IncK, IncQ, IncR, IncX, IncY groups and not predominantly in IncF type plasmids (Rozwandowicz et al., 2018). The presence of IncI-gamma/K1 plasmids is not surprising as these are predominantly found in Europe, carrying tetracycline resistance genes (Pezzella et al., 2004; Rozwandowicz et al., 2018). Dissemination of *mcr* genes, conferring resistance to colistin, was first detected on IncHI2 plasmid which was also the case in our strains. In addition, the *mcr-2.1* gene was identified on a IncX4 plasmid (Falgenhauer et al., 2016; Boueroy et al., 2022). This plasmid was first identified in Denmark and is a global concern as elevated numbers of human plasmids were identified to carry colistin resistance genes (Hasman et al., 2015; Elbediwi et al., 2019). Increased resistance to florfenicol is also worrying as this antimicrobial drug is commonly used on farms. The rapid spread of florfenicol-associated genes, including *floR*, was shown to be mediated through various MGEs posing the potential co-dissemination of tetracycline and aminoglycoside ARGs (Yang et al., 2022). In our dataset, plasmids were identified which carry the *floR* gene (IncFIA or IncY rep types). This *floR*-carrying IncFIA plasmid also showed the presence of 11 other ARGs, including genes against tetracycline and aminoglycosides. The latter were shown to be present on IncF plasmids among others. Our data showed its presence predominantly on IncF, next to IncX1 and IncI-gamma/K1 plasmids, along with various multi-replicon plasmids.

In conclusion, our data highlights the feasibility to accurately infer information on bacterial identification, virotyping, and AST from genetic information in the context of enteric colibacillosis. This will allow quicker and cost-efficient ETEC/STEC diagnostics as compared to *default* combined culture-based and multiplex PCR methods. Even though the current method relies on purified ETEC/STEC cultures, this knowledge can be exploited in *all-in-one* (meta)genomic workflows which are promising to revolutionize current routine diagnostics (Theuns et al., 2018; Leggett et al., 2020b; Bokma et al., 2021b; Khan et al., 2022; Yang et al., 2022). The immediate availability of all this data will contribute to a better understanding of pathogen epidemiology and dissemination of virulence and ARGs. Furthermore, it will support practitioners to quickly initiate tailored treatments or address management changes in a more cost-efficient manner.

## Data availability statement

The datasets presented in this study can be found in online repositories. The names of the repository/repositories and accession number(s) can be found at: https://www.ncbi.nlm.nih.gov/, PRJNA917806.

## Author contributions

NV and SV: conceptualization, validation, formal analysis, and data curation. NV, SV, and ST: methodology. NV: software, investigation, visualization, writing—original draft preparation, and project administration. BD, EC, and ST: resources. NV, BD, and ST: writing—review and editing. ST and BD: supervision. NV, BD, EC, and ST: funding acquisition. All authors have read and agreed to the published version of the manuscript.

## Funding

NV was funded by a grant from the Flemish Agency for Innovation and Entrepreneurship (Baekeland Mandate HBC.2020.2889).

## Conflict of interest

ST is co-founder and NV is an employee at PathoSense BV. DS is employed at CEVA animal health.

The remaining authors declare that the research was conducted in the absence of any commercial or financial relationships that could be construed as a potential conflict of interest.

## Publisher’s note

All claims expressed in this article are solely those of the authors and do not necessarily represent those of their affiliated organizations, or those of the publisher, the editors and the reviewers. Any product that may be evaluated in this article, or claim that may be made by its manufacturer, is not guaranteed or endorsed by the publisher.

## Footnotes

^1^ https://github.com/rrwick/Filtlong

^2^ https://github.com/rrwick/Minipolish

^3^ https://github.com/ablab/viralVerify

^4^ https://github.com/tseemann/abricate

^5^ https://github.com/phac-nml/ecoli_vf
