# Supplementary figures and images for: Virotyping and genetic antimicrobial susceptibility testing of porcine ETEC/STEC strains and associated plasmid types
Source: Front Microbiol. 2023 Apr 17;14:1139312. doi: 10.3389/fmicb.2023.1139312 (PMC10151945; doi:10.3389/fmicb.2023.1139312)

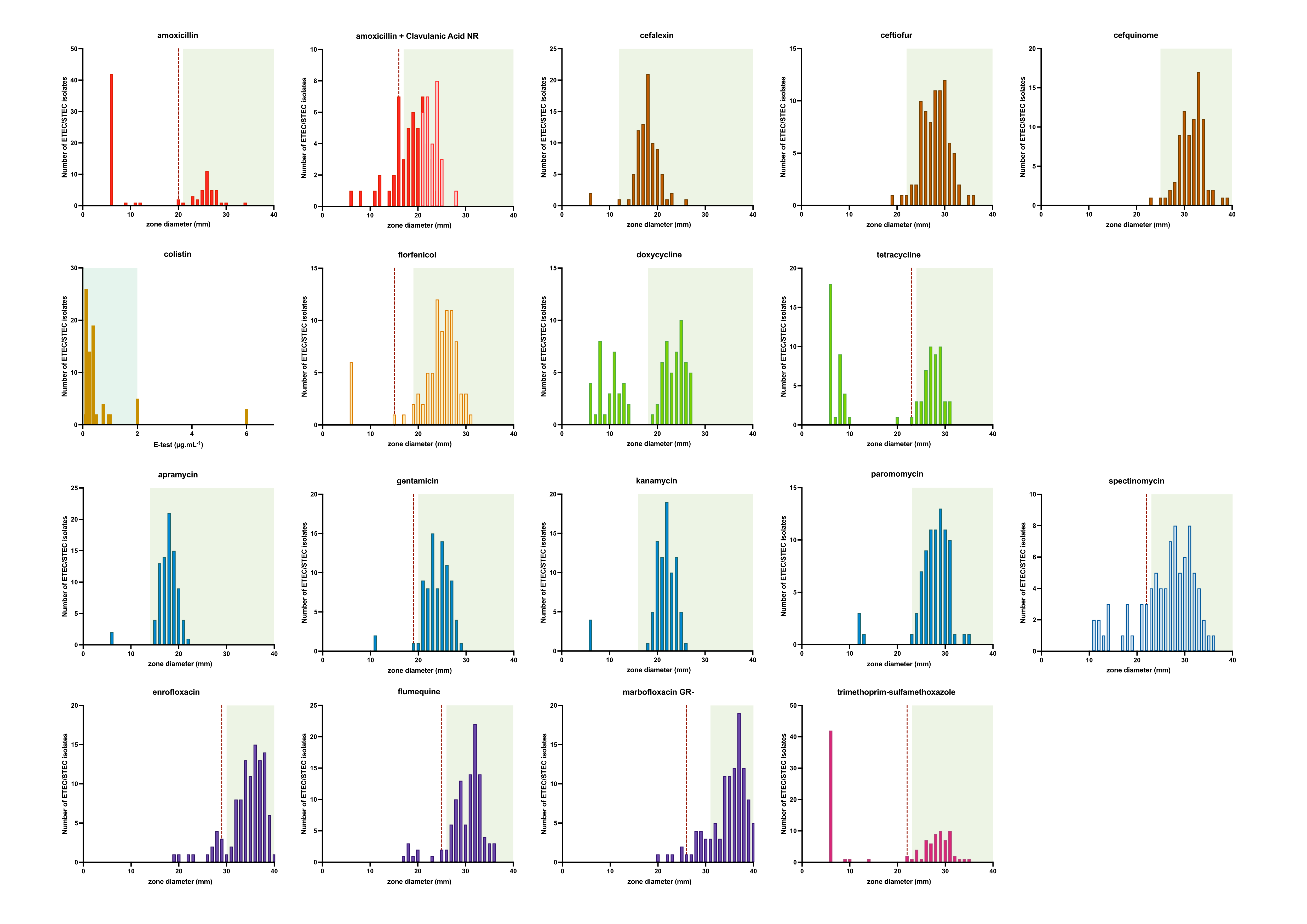

Supplement: Supplementary file 6 [file Image_1.TIFF]
